# Multiple drilling combined with simvastatin versus multiple drilling alone for the treatment of avascular osteonecrosis of the femoral head: 3-year follow-up study

**DOI:** 10.1186/s12891-016-1199-0

**Published:** 2016-08-15

**Authors:** Han Yin, Zhenfeng Yuan, Dawei Wang

**Affiliations:** Department of Orthopaedics, Liaocheng People’s Hospital and Liaocheng Clinical School of Taishan Medical University, No. 67, Dongchang Road, Liaocheng, Shandong China

**Keywords:** Femoral head necrosis, Core decompression, Multiple decompression, Simvastatin, Preservation of hip

## Abstract

**Background:**

Multiple small drilling for core decompression is widely used to preserve the femoral head in patients with avascular necrosis of the femoral head (ANFH). Nevertheless, the clinical outcome remains controversial. Simvastatin has been demonstrated to promote bone formation and reduce bone adsorption. The purpose of this study was to determine whether simvastatin enhanced the effect of multiple decompressions in preventing progression of ANFH and to identify independent risk factors associated with poor results.

**Methods:**

We retrospectively analyzed 58 hips in 36 patients, with a follow-up of 36 months. 20 patients (32 hips) underwent multiple drilling combined with simvastatin treatment (SIM group); 16 patients (26 hips) underwent multiple drilling alone (MD group). We defined clinical failure as a requirement for subsequent hip surgery or Harris Hip Score < 75. New occurrence of collapse or increased collapse > 2 mm on plain radiographs was defined as radiological failure.

**Results:**

Successful clinical results were achieved in 27 of 32 hips (84 %) in the SIM group compared with 15 of 26 hips (58 %) in the MD group (OR = 0.2, CI (0.1, 0.6.), *P* = 0.032). Successful radiological results were achieved in 27 of 32 hips (84 %) in the SIM group and in 16 of 26 hips (61.5 %) in the MD group (*P* = 0.048). Body mass index, disease stage and location of lesion were independent prognostic factors for overall survival.

**Conclusions:**

We believe that simvastatin could enhance the effects of multiple decompressions in preventing progression of ANFH and reducing the risk of femoral head collapse.

## Background

Avascular necrosis of the femoral head (ANFH) is a common cause of hip disability in relatively young, active people between 20 and 40 years of age. It may progress to collapse of the femoral head if left untreated [1] and 80 % of these patients will require total hip arthroplasty (THA) [2]. Preservation of the femoral head is the ultimate goal in the treatment of ANFH [3]. Although many treatment methods for early stage ANFH, including electrical stimulation, core decompression, rotational osteotomy and nonvascularized and vascularized bone grafting, have been proposed based on patient age, symptoms, stage, and/or medical status, the orthopedic community has not yet adopted a uniform treatment algorithm [4].

Core decompression, which is thought to decrease intramedullary pressure, encourage revascularization and relieve pain, is a widely accepted procedure for early stage ANFH [5, 6]. Although core decompression and core decompression in combination with bone graft, bone marrow injection, platelet-rich plasma injection or mesenchymal stem cell injection have achieved excellent clinical outcomes in the treatment of ANFH [2, 5, 7, 8], some reports note complication rates as high as 10–15 % [8–11]. Seeking a minimally invasive, safe and effective treatment for ANFH, Kim et al. proposed the use of multiple small drilling for core decompression at the annual Association Internationale de Recherche sur la Circulation Osseuse (ARCO) meeting in 2003. They reported a lower rate of collapse (14.3 %) compared with traditional core decompression methods (45 %) 3 years after surgery [12].

Statins have been demonstrated to reduce the risk of corticosteroid-induced ANFH through improving disturbances of lipid metabolism [13–16]. In addition, it has been reported that statins can increase the expression of bone morphogenetic protein 2 (BMP-2) mRNA in osteoblasts, promote bone formation [17, 18] and decrease the formation and activity of osteoclasts, inhibiting bone resorption [19, 20]. Clinical studies have discovered that statins can reduce fracture risk [21], increase bone mineral density [22] and promote the expression of biochemical markers of bone metabolism to serve as a tool for the diagnosis of osteoporosis [23].

The purpose of the present study was to: (1) determine whether simvastatin can enhance the effects of multiple decompressions in preventing the progression of ANFH and reducing the risk of femoral head collapse; and (2) identify independent risk factors associated with poor results.

## Methods

**Fig. 1:**
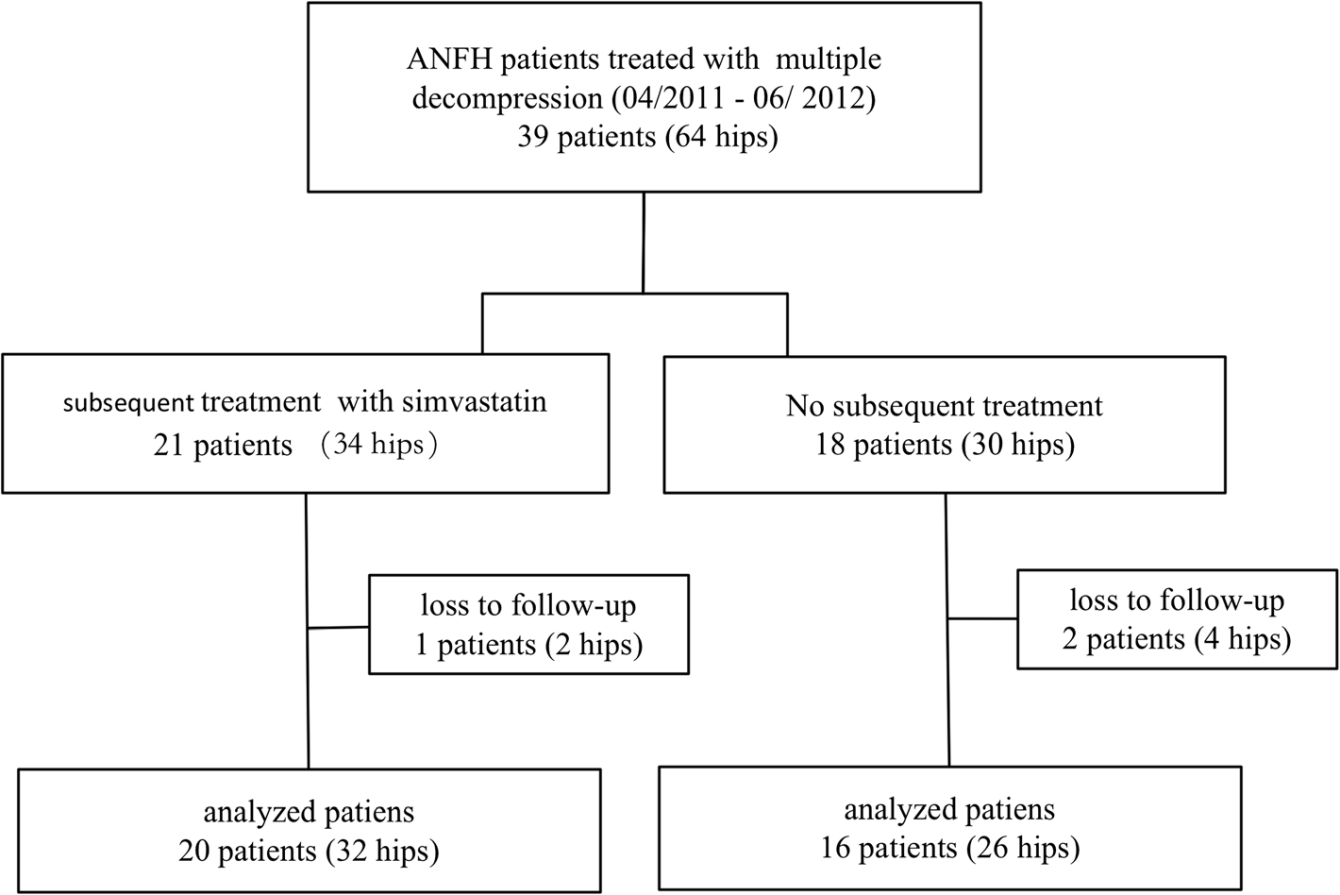
Flow diagram of patient selection. ANFH, avascular necrosis of femoral head

Multiple small-diameter drilling was performed by one of us (Zhenfeng Yuan) between April 2011 and June 2012 in 39 patients (64 hips) at the same institution for the treatment of non-traumatic osteonecrosis of the femoral head. Of these patients, 21 (34 hips) received simvastatin after the procedure. Eighteen patients (30 hips) who refused subsequent treatment with simvastatin were taken as controls. Patients in the MD group underwent core decompression with multiple small-diameter drilling. Patients in the SIM group underwent multiple drilling combined with oral simvastatin, 20 mg per day for 12 months beginning on the day after the drilling procedure. In the MD group, two patients (four hips) were lost to follow-up; therefore, 16 patients (26 hips) were available for study. In the SIM group, one patient (two hips) was lost to follow-up; therefore, 20 patients (32 hips) were available for study (Fig. 1). Observations were truncated 36 months postoperatively to permit statistical comparison between the two groups. This study was approved by the ethics committee of Liaocheng People’s Hospital and informed consent was obtained from all individual participants included in the study.

Osteonecrosis of the femoral head was diagnosed if magnetic resonance imaging (MRI) (coronal and sagittal) revealed the following: belt-shaped or circular low intensity signals surrounded by high intensity signals in the outer area on short tau inversion; or a high intensity area surrounded by belt-shaped or circular low intensity signals within the femoral head on T1-weighted images [24]. The patients were classified according to the ARCO system [25]. Patients were eligible for inclusion in the study if they were older than 18 years, had hip pain and had ARCO stage I, II or IIIA ANFH. Patients were excluded from the study if they had ARCO stage IIIB, IIIC or IV ANFH. The procedure was not performed on patients older than 60 years.

The largest anteroposterior diameter of the head (R), the longest anteroposterior length of the necrotic lesion (A) and the longest mediolateral length of the necrotic lesion (B) were measured on axial slices on MRI. The extent of the necrotic portion was calculated by the equation: percentage of necrotic lesion = {(A × B)/R^2^} × 100. This method has a more acceptable accuracy and is reasonably repeatable compared with other methods [26]. All hips were assessed using this method, with necrotic portions greater than 30 % considered large lesions, 15–30 % medium lesions and less than 15 % small lesions. The location of the necrotic lesion was classified as A, B, C1 or C2 according to the classification developed by the Specific Disease Investigation Committee under the auspices of the Japanese Ministry of Health, Labor and Welfare [27]. Three experienced radiologists reviewed all MRI scans and plain radiographs together in a blinded fashion.

Before the procedure, details of the patient’s age, gender and body mass index (BMI), duration of symptoms, cause of ANFH, unilateral or bilateral involvement, ARCO stage, lesion size, location of lesion, Harris Hip Score (HHS) and visual analog scale (VAS) pain score were recorded (Table 1).

**Table 1:** Baseline patient characteristics

Characteristic	Total	SIM Group	MD Group	*P* value
Sex				0.479
Men	26 (72)	13 (65)	13 (81)	
Women	10 (28)	7 (35)	3 (19)	
Age (years)				
Mean	41.6 ± 1.1	39.1 ± 1.4	44.7 ± 1.6	0.010
Median	42 (37.8–48)	40 (36–44.5)	45 (41.8–49)	0.003^a^
BMI (kg/m^2^)				
Mean	25.6 ± 0.3	25.6 ± 0.4	25.6 ± 0.6	0.958
Median	25.2 (23.7–26.7)	24.7 (23.5–26.7)	25.3 (23.7–26.5)	0.833^a^
Duration of symptoms (months)	3 (1–6.5)	3 (1.13–8)	2.5 (1–5.25)	0.354
Laterality				0.878
Unilateral	14 (39)	8 (40)	6 (37)	
Bilateral	22 (61)	12 (60)	10 (63)	
Left: Right	29:29	16:16	13:13	1.000
Cause of ANFH				0.640^b^
Steroids	6 (10)	3 (9)	3 (11)	
Alcohol	24 (41)	15 (47)	9 (35)	
Idiopathic	28 ( )	14 (44)	14 (54)	
ARCO stage				0.108^b^
I	10 (17)	3 (9)	7 (27)	
II a	2 (3)	1 (3)	1 (4)	
II b	12 (21)	10 (32)	2 (8)	
II c	29 (50)	16 (50)	13 (50)	
IIIa	5 (9)	2 (6)	3 (11)	
Lesion size				0.021^b^
Small	8 (14)	2 (6)	6 (23)	
Medium	13 (22)	11 (34)	2 (8)	
Large	37 (64)	19 (60)	18 (69)	
Lesion location				0.770^b^
A	2 (3)	2 (6)	0 (0)	
B	26 (45)	14 (44)	12 (46)	
C1	15 (26)	8 (25)	7 (27)	
C2	15 (26)	8 (25)	7 (27)	
Hydrarthrosis				0.985
Yes	20 (34)	11 (34)	9 (25)	
No	38 (66)	21 (66)	17 (75)	
VAS	6.1 ± 1.2	6.2 ± 1.4	6.0 ± 1.1	0.546
HSS score	66 ± 5.2	65 ± 5.7	68 ± 4.5	0.767

^a^Determined with the Mann–Whitney *U* test

^b^Determined with the Fisher exact test

Patients were followed-up 1, 3, 6, 9, 12, 24 and 36 months after the procedure. In addition, they received a follow-up visit whenever they were unwell. Follow-up took the form of outpatient visits. Examinations included joint pain, function and range of motion. HHS and VAS pain score were recorded. Plain radiographs (anteroposterior and frog lateral view) and MRI scans of both hips were obtained at scheduled follow-up times. The primary outcomes of the study were clinical and radiological failure. Clinical failure was defined as HHS < 75 points or a requirement for subsequent hip surgery such as bone grafting, osteotomy or hip replacement. New occurrence of collapse or increased collapse of greater than 2 mm on plain radiographs during follow-up was defined as radiological failure [28, 29].

For the drilling procedure, patients were placed in the supine position on a fracture table. A C-arm fluoroscope was draped with a sterile sleeve and positioned over the hip region to enable an anteroposterior view. After the position of the femoral head had been marked and the hip draped, a 3.0 mm Steinman pin was inserted percutaneously under fluoroscopic guidance. The pin was inserted into the femoral head at the site of the lesion. Anteroposterior and lateral fluoroscopic views were necessary while advancing the pin to ensure that it remained in the medullary canal of the femoral neck. Depending on lesion size, each femoral head was drilled three to six times. The wound was closed using a simple bandage without suture.

Patients who had undergone unilateral drilling were advised to use two crutches when walking for 6 weeks after the procedure. Two crutches when walking for 12 weeks were advised for patients who had undergone a bilateral procedure. Patients were allowed to engage in physical activities and sports 12 months after surgery. No further rehabilitation program was implemented [28].

Statistical analysis of the data was performed using SPSS version 18.0 for Windows (IBM, Armonk, NY, USA). Baseline characteristics were presented using descriptive statistics. The chi-square test was used to compare nominal data. The *t*-test or Mann–Whitney *U* test was used to compare metric data. Univariate analyses were performed using the chi-square test. Variables with a *P* value of less than 0.10 on univariate analysis were entered into multivariate analysis. Causes of ANFH were also entered into multivariate analysis. Multivariate analysis was performed using a logistic proportional hazards regression model. Because of the multicollinearity between ARCO stage and lesion size, models were fitted using the forward conditional procedure. All statistical assessments were two-sided and evaluated at the 0.05 level of statistical significance.

## Results

Detailed baseline patient characteristics are shown in Table 1. Patients in the SIM group were younger (mean age 40 years, range 36–44.5) than those in the MD group (mean age 45 years, range 41.8–49) (*P* = 0.003). There were more patients with medium or large necrotic lesions in the SIM group than in the MD group (*P* = 0.021).

**Fig. 2:**
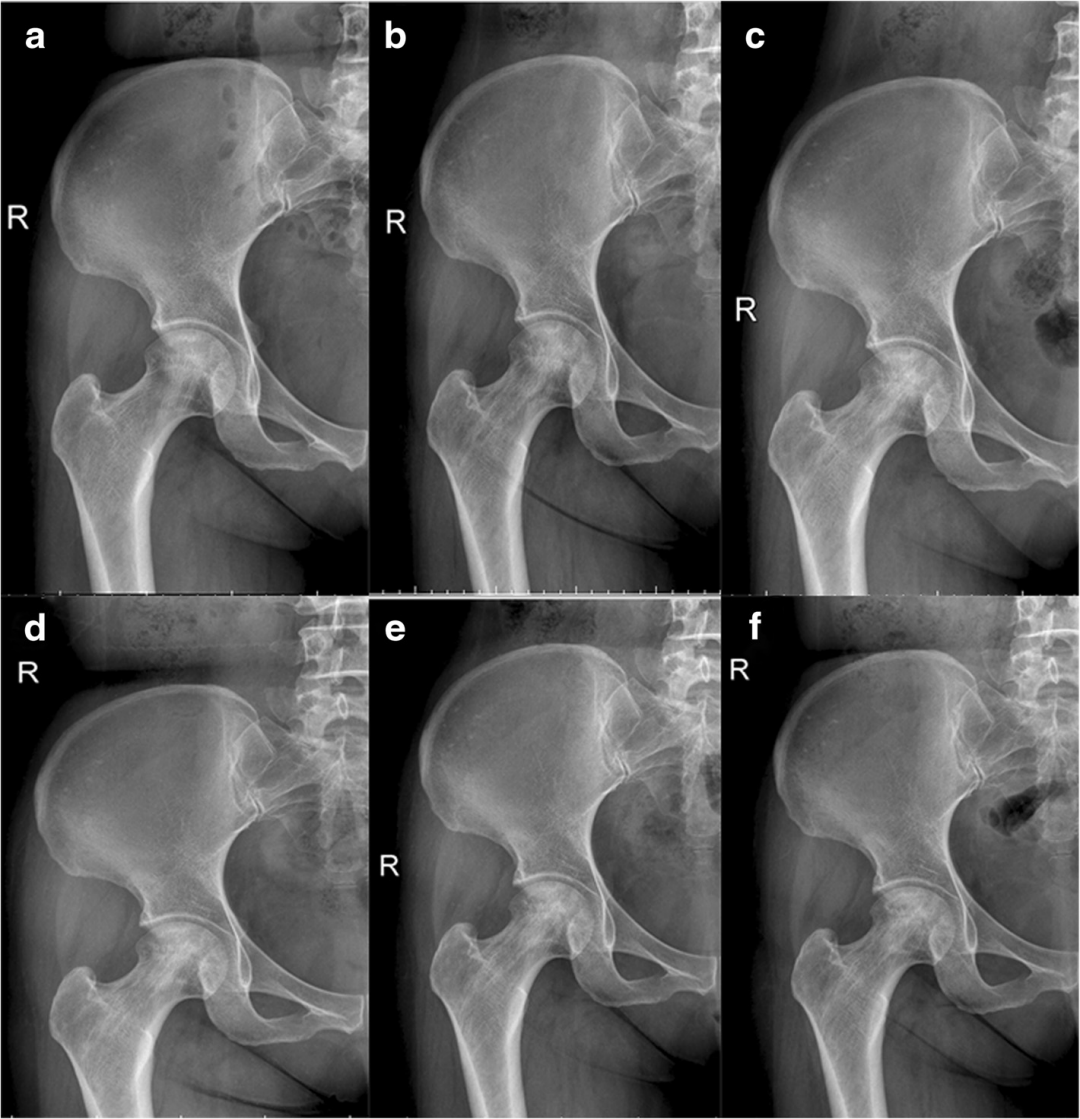
Representative radiographs from SIM group. **a** Preoperative; **b**–**f** 3, 9, 12, 24 and 36 months postoperatively

At 36 months follow-up, the proportion of successful clinical results was significantly higher in the SIM group compared with the MD group (*P* = 0.024). Successful clinical results were achieved in 27 of 32 hips (84 %) in the SIM group (Fig. 2). Of the five hips (16 %) that were considered clinical failures, two underwent THA because of secondary degenerative arthritis 18 months and 26 months after the drilling procedure; the other three had not undergone any further reconstructive procedures at the time of the last follow-up visit. In the MD group, successful clinical results were achieved in 15 of 26 hips (58 %). Of those that were clinical failures, seven hips underwent THA because of secondary degenerative arthritis at a mean of 14 months (range 6–26) after the procedure, one underwent vascularized fibular grafting at 15 months and the remaining three had not undergone any further reconstructive procedures at the last follow-up. The SIM group had a better radiological outcome than the MD group (*P* = 0.048). Successful radiological results were achieved in 27 of 32 hips (84 %) in the SIM group and in 16 hips (61.5 %) in the MD group (Table 2).

**Table 2:** Univariate analysis of prognostic factors for clinical success rate

	Total	Success	Failure	*P* value
Treatment				0.024
SIM	32	27 (84)	5 (16)	
MD	26	15 (58)	11 (42)	
Age (years)				0.443
< 40	17	14 (82)	3 (18)	
> 40	41	28 (68)	13 (32)	
Sex				0.955
Men	42	31 (74)	11 (26)	
Women	16	11 (69)	5 (31)	
BMI (kg/m2)				0.019
< 25	29	25 (86)	4 (14)	
≥ 25	29	17 (59)	12 (41)	
Duration of symptoms (months)				0.951
< 3	25	18 (72)	7 (28)	
> 3	33	24 (73)	9 (27)	
Laterality				1.000
Unilateral	14	10 (71)	4 (29)	
Bilateral	44	32 (73)	12 (27)	
Cause of ANFH				0.764^a^
Steroids	6	5 (83)	1 (17)	
Alcohol	24	18 (75)	6 (25)	
Idiopathic	28	19 (68)	9 (32)	
ARCO stage				0.001
I, II a, II b	24	23 (96)	1 (4)	
II c,III a	34	19 (56)	15 (44)	
Lesion size				0.007^a^
Small + Medium	21	21 (100)	0 (0)	
Large	37	21 (57)	16 (43)	
Lesion location				0.029
A, B	28	24 (86)	4 (14)	
C1, C2	30	18 (60)	12 (40)	
Hydrarthrosis				0.031
Yes	20	11 (55)	9 (45)	
No	38	31 (82)	7 (18)	

^a^Determined with the Fisher exact test

Percentages are in parentheses

There was a significant difference between the preoperative and last follow-up HHS in both groups and a significant difference was observed between the groups in the last follow-up HHS (88.6 ± 7.3 vs 79.4 ± 5.3, *P* = 0.034). A significant difference was observed between the SIM group (2.4 ± 0.8) and the MD group (3.6 ± 0.6) in the last follow-up VAS score (*P* = 0.014).

The procedure was a clinical success in nine of the 10 stage I hips (90 %), both stage IIa (100 %), all 12 stage IIb (100 %), 18 of the 29 stage IIc (62.1 %) and one of the five stage IIIa (20 %). When the hips were divided into earlier stage (ARCO stage I, IIa or IIb) and later stage (ARCO stage IIc or IIIa), 23 of the 24 (96 %) earlier stage hips had successful clinical results compared with 19 of the 34 (56 %) later stage hips (*P* = 0.001) (Table 2). When the hips were stratified by lesion size, all (100 %) hips with a small or medium necrotic lesion had successful clinical results compared with only 21 of the 37 (57 %) hips with a large lesion (*P* = 0.007) (Table 2). Among hips (52) with non-corticosteroid-induced ANFH, 24 of the 29 (82.8 %) hips in the SIM group had successful clinical results compared with 13 of the 26 (50 %) hips in the MD group (*P* = 0.038) (Table 2).

On univariate analysis, lesions located medially, low BMI and no hydrarthrosis were associated with significantly better clinical outcome (*P* = 0.029, *P* = 0.019 and *P* = 0.031) (Table 2). Treatment type, ARCO stage, necrotic lesion size, location of lesion, BMI and hydrarthrosis were included in multivariate analysis. Cause of ANFH (corticosteroids, alcohol or idiopathic) has been considered a predictor of clinical success in many studies [8, 29]; therefore, we also included cause of ANFH in our multivariate analysis, although it was not a predictor of clinical success on univariate analysis in our patient population. On multivariate analysis, treatment type, BMI, ARCO stage and lesion location were identified as independent prognostic factors for overall survival (Table 3).

**Table 3:** Multivariate analysis of prognostic factors for clinical success rate

	Odd ratio^a^	*P* value^b^
Treatment		0.032
MD	1	
SIM	0.2 (0.1,0.6)	
BMI (kg/m2)		0.014
< 25	1	
≥ 25	20.9 (1.9,234.1)	
ARCO stage		0.006
I, II a, II b	1	
II c,III a	32.1 (3.4,530.4)	
Lesion location		0.018
A, B	1	
C1, C2	17.4 (1.6,185.9)	

^a^Data in parentheses are 95 % CIs

^b^Determined with logistic regression analysis

There were no superficial infections, deep infections, femoral neck fractures, subtrochanteric fractures, heterotopic ossifications, hematomas or other complications associated with the procedure in either group. No patient has simvastatin-related complications.

## Discussion

To the best of our knowledge, this is the first study comparing the beneficial effects on ANFH of systemic simvastatin following core decompression by multiple small-diameter drilling. Our preliminary results show that multiple small-diameter drilling combined with systemic simvastatin was better than multiple small-diameter drilling alone.

Given the relatively young age of patients at the time of presentation and the fact that the currently available prostheses may not be used for a lifetime, the treatment goal for early stage ANFH is to preserve the femoral head rather than replace it [30]. Multiple small-diameter drilling has been widely employed for the treatment of ANFH since Kim et al. proposed its use in 2003; however, its efficacy remains controversial (Table 4), with 30–40 % of patients suffering clinical failure [6, 28, 31]. To improve the clinical outcome of multiple small drilling for core decompression, several adjunctive methods have been added following the procedure. Kang et al. administered systemic alendronate as a femoral head-preserving method in ANFH; the clinical success rates in patients with stage II or stage III disease increased by 11.5 and 15.3 %, respectively, at a minimum of 4 years follow-up [29]. However, in research conducted by Lim et al., stem cell implantation after multiple drilling did not improve the clinical outcome compared with the conventional method of core decompression (57 % vs 54.8 %) at 5-year follow-up [32]. In this study, systemic administration of simvastatin after multiple drilling significantly improved the overall clinical success rate from 57.7 to 84.4 %.

**Table 4:** Literature review

Study	Treatment	Success rate			Follow-up (months)	Overall success rate
		I	II	III		
Song et al.	Multi-drilling CD	79.5 %	76.6 %	34.9 %	87 (60–134)	66.3 %
Mont et al.	Multi-drilling CD	80 %	57 %	-	24 (20–39)	71 %
Kang et al.	Multi-drilling CD	-	79 %	46.2 %	62 (49–71)	71.2 %
Lee et al.	Multi-drilling CD	100 %	65 %	40 %	37.1 (24–60)	56 %
Our research	Multi-drilling CD	85.7 %	56.2 %	-	36	57.7 %
Omran et al.	Multi-drilling CD	100 %	66.7 %	-	At least 24	72.7 %
Kang et al.	Multi-drilling CD + alendronate	-	90.5 %	61.5 %	63 (48–75)	83.6 %
Lim et al.	Multi-drilling CD + stem cell	-	60.8 %	42.9 %	60	53.9 %
Our research	Multi-drilling CD + simvastatin	100 %	82.6 %	-	36	81.5 %

Statins inhibit hydroxymethylglutaryl-coenzyme A reductase, one of the rate-limiting enzymes of the mevalonate pathway, and are widely used for the treatment of hyperlipemia. Recently, statins have been demonstrated to be effective for the prophylaxis of corticosteroid-induced ANFH through counteracting the effects of corticosteroid-induced adipogenesis in bone stem cells and systemic changes in lipid metabolism [14, 16, 33, 34]. However, until now it was unknown whether statins can improve the clinical outcome in non-corticosteroid-induced ANFH. Our results show that simvastatin might increase the clinical success rate in non-steroid induced ANFH. There may be several possible mechanisms for this effect. (1) Promotion of bone formation: By inhibiting the mevalonate pathway and preventing the prenylation and function of small GTPases, BMP-2 expression may be stimulated, causing increased osteoblast expression and differentiation and subsequent enhancement of bone formation [35]. (2) Inhibition of bone absorption: By suppressing cellular membrane fusion events [36] and the RANKL signaling system [19], formation of osteoclasts could be inhibited and, by inhibiting of the mevalonate pathway [37], osteoclast function might be affected. (3) Recovery of blood supply: Simvastatin could promote the proliferation of vascular endothelial cells through stimulating the release of vascular endothelial growth factor, promoting recovery of the blood supply to the necrotic area [38].

Several studies have demonstrated that the clinical outcome of osteonecrosis of the femoral head is associated with the stage of the disease [8, 28, 29]. Early disease has a better clinical outcome. In agreement with these studies, we achieved excellent results in hips with earlier stage disease. Our results confirm that BMI can serve as a valuable parameter for the evaluation of clinical outcome. Patients with a higher BMI might have a more rapidly progressive form of the disease. Although several studies have suggested that clinical outcomes are worse in patients with corticosteroid-related osteonecrosis [8, 39, 40], in the present study the clinical outcome was unrelated to corticosteroid use. Few hips with corticosteroid-related osteonecrosis in this study might explain this discrepancy.

Our study has several limitations. First, this was a retrospective study with a small number of individuals. A large scale randomized controlled trial is essential to evaluate the clinical efficacy of the treatment. Second, the follow-up period was short, with observations truncated 36 months postoperatively. Studies show that most clinical failures occur within 3 years postoperatively [8, 11], but a longer follow-up period will still be needed in the future. Third, the simvastatin regimen was chosen according to that used to treat osteoporosis [41]. The optimal dose and duration of simvastatin treatment remain to be determined.

## Conclusion

Despite these shortcomings, from the results of our research it seems reasonable to assume that simvastatin can enhance the effects of multiple decompressions in preventing the progression of ANFH and reducing the risk of femoral head collapse. Long term results and greater numbers of patients are needed to make definitive conclusions.

## Abbreviations

ANFH, Avascular necrosis of the femoral head; ARCO, Association Internationale de Recherche sur la Circulation Osseuse; BMI, Body mass index; BMP-2, Bone morphogenetic protein 2; HHS, Harris Hip Score; MRI, Magnetic resonance imaging
